# Dynamic APACHE II Score to Predict the Outcome of Intensive Care Unit Patients

**DOI:** 10.3389/fmed.2021.744907

**Published:** 2022-01-26

**Authors:** Yao Tian, Yang Yao, Jing Zhou, Xin Diao, Hui Chen, Kaixia Cai, Xuan Ma, Shengyu Wang

**Affiliations:** Department of Pulmonary and Critical Care Medicine, The First Affiliated Hospital of Xi'an Medical University, Xi'an, China

**Keywords:** APACHE II, mortality, intensive care units, MIMIC-IV, predictor

## Abstract

**Objective:**

This study aims to evaluate the accuracy of the Acute Physiology and Chronic Health Evaluation (APACHE) II score on different days in predicting the mortality of critically ill patients to identify the best time point for the APACHE II score.

**Methods:**

The demographic and clinical data are retrieved from the Medical Information Mart for Intensive Care (MIMIC)-IV dataset. APACHE II scores on days 1, 2, 3, 5, 7, 14, and 28 of hospitalization are calculated, and their performance is evaluated using the area under the receiver operating characteristic (AUROC) analysis. The cut-off for defining the high risk of mortality is determined using Youden's index. The APACHE II score on day 3 is the best time point to predict hospital mortality of ICU patients. The Hosmer-Lemeshow goodness-of-fit test is then applied to evaluate the calibration of the day 3 APACHE II score.

**Results:**

We recruited 6,374 eligible subjects from the MIMIC-IV database. Day 3 is the optimal time point for obtaining the APACHE II score to predict the hospital mortality of patients. The best cut-off for day 3 APACHE II score is 17. When APACHE II score ≥17, the sensitivity for the non-survivors and survivors is 92.8 and 82.2%, respectively, and the positive predictive value (PPV) is 23.1%. When APACHE II socre <17, the specificity for non-survivors and survivors is 90.1 and 80.2%, respectively, and the negative predictive value (NPV) is 87.8%. When day-3 APACHE II is used to predict the hospital mortality, the AUROC is 0.743 (*P* <0.001). In the ≥17 group, the sensitivity of non-survivors and survivors is 92.2 and 81.3%, respectively, and the PPV is 30.3%. In the <17 group, the specificity of non-survivors and survivors is 100.0 and 80.2%, respectively, and the NPV is 81.6%. The Hosmer-Lemeshow test indicated day-3 APACHE II has a high predicting the hospital mortality (*X*^2^ = 6.198, *P* = 0.625, consistency = 79.4%). However, the day-1 APACHE II has a poor calibration in predicting the hospital mortality rate (*X* ^2^ = 294.898, *P* <0.001).

**Conclusion:**

Day-3 APACHE II score is an optimal biomarker to predict the outcomes of ICU patients; 17 is the best cut-off for defining patients at high risk of mortality.

## Introduction

Predicting the mortality of patients in ICU plays an important role in patient care and resource allocation. Early identification and management of patients at a higher risk of mortality are associated with lower mortality rates (1, 2). In recent years, several scoring systems have been developed to evaluate the severity of illness and predict the outcomes (3–5), especially the mortality (6, 7) of critically ill patients, such as Acute Physiology Chronic Health Evaluation II (APACHE II), Organ Dysfunction and Infection System, Sequential Organ Failure Assessment (SOFA) and Simplified Acute Physiology Score (SAPS). Among these severity-of-disease classification systems, the APACHE II score is the most commonly used method, which generates a point score ranging from 0 to 71 based upon initial values of 12 acute physiologic variables, age, and previous health status. A higher score corresponds to more severe disease and a higher risk of death. Currently, most studies use the APACHE II score within 24 h after hospitalization to predict the outcomes of patients (2, 8). However, in the first 24 h, many patients have complex comorbid conditions, thus selecting only one principal diagnostic category may not be plausible. Moreover, the condition of patients may be unstable, and the physiological indexes may fluctuate greatly in the first several days of hospitalization. The treatments may also lead to significant changes in the conditions and outcomes of the patients. These factors raise concerns that APACHE II score within 24 h may not be able to accurately predict the outcomes and mortality of critically ill patients.

In this study, we evaluated the accuracy of APACHE II scores on different days in predicting the outcomes of a large cohort of ICU patients. We found that the third-day APACHE II score is the optimal biomarker to predict the outcomes of ICU patients, and 17 is the best cut-off for defining patients at higher risk of mortality.

## Materials and Methods

### Date Source

The data used in this study were retrieved from the Medical Information Mart for Intensive Care (MIMIC)-IV database, a large, publicly available database comprising the clinical diagnosis and treatment information of ~53,150 critically ill patients admitted to the Beth Israel Deaconess Medical Center (Boston, Massachusetts, USA) from 2008 to 2019 (9–11). We obtained the approval to access the database (Certification Number: 36379199) after completing the National Institutes of Health web-based training course “Protecting Human Research Participants.”

### Data Extraction and Management

The following data were extracted from the database: age, sex, survival status, admission type, date of ICU and hospital admission and discharge, date of birth and death, and the hospital and ICU length of stay (LOS). Glasgow Coma Scale (GCS), physiological variables, and laboratory results. Data were extracted from the following tables: ADMISSIONS, ICUSTAYS, PATIENTS, CHARTEVENTS, LABARARY EVENTS, and DIAGNOSIS_ICD in the database. Patients discharged from the hospital or deceased before day 28 were censored to the last known APACHE II score. APACHE II score system included 12 physiological variables (temperature, mean arterial pressure, heart rate, respiratory rate, A-a PO_2_ (Fio_2_ > 50%) or PaO_2_ (Fio_2_ <50%), arterial PH or HCO_3_, serum sodium, potassium and creatinine, hematocrit, white blood cell count and GCS), a chronic health evaluation and age adjustment score. Each variable is weighted from 0 to 4, and the range of the total Apache II score is from 0 to 71 points. Two authors independently extracted the relevant data and assessed the eligibility and quality of the study. The days of hospitalization were calculated by subtracting the date of admission from the date of discharge. The data were de-identified to ensure the privacy of patients (12). Therefore, the days of death after discharge were calculated by subtracting the date of discharge from the date of death, and the 90-day mortality rate was further calculated. The disease classification was gradually refined according to the *International Classification of Diseases-9th* (ICD-9) edition comorbidities (13). APACHE II scores of the recruited patients on days 1, 2, 3, 5, 7, 14, and 28 after hospitalization in ICU were calculated as described in previous studies (14, 15). The primary endpoint was hospital mortality; the secondary endpoint was 90-day mortality. For patients admitted to ICU more than once, only the first ICU admission was included. Patients were excluded if (1) they stayed in ICU <1 day; (2) they have missing data of APACHE II score; (3) they were under the age of 18 when admitted to ICU.

### Statistical Analysis

All statistical analyses were performed using the IBM SPSS Statistics software (IBM, Armonk, NY, USA). Quantitative and categorical variables were expressed as mean ± standard deviation (S.D.) and number and percentage, respectively. The means of two groups were compared using the *t*-test if the data were normally distributed and the variance was homogeneous. If not, data were represented by the median and interquartile range and compared by the Wilcoxon rank-sum test. Discrimination and calibration were assessed using the area under the ROC (AUROC) and Hosmer-Lemeshow statistic, respectively. The Youden index was used to identify the optimal cut-off value for defining patients at a higher risk of mortality. A *P* < 0.05 was considered statistically significant.

## Results

### Demographic Characteristics, APACHE II Scores, and Outcomes of Patients

**Figure 1:**
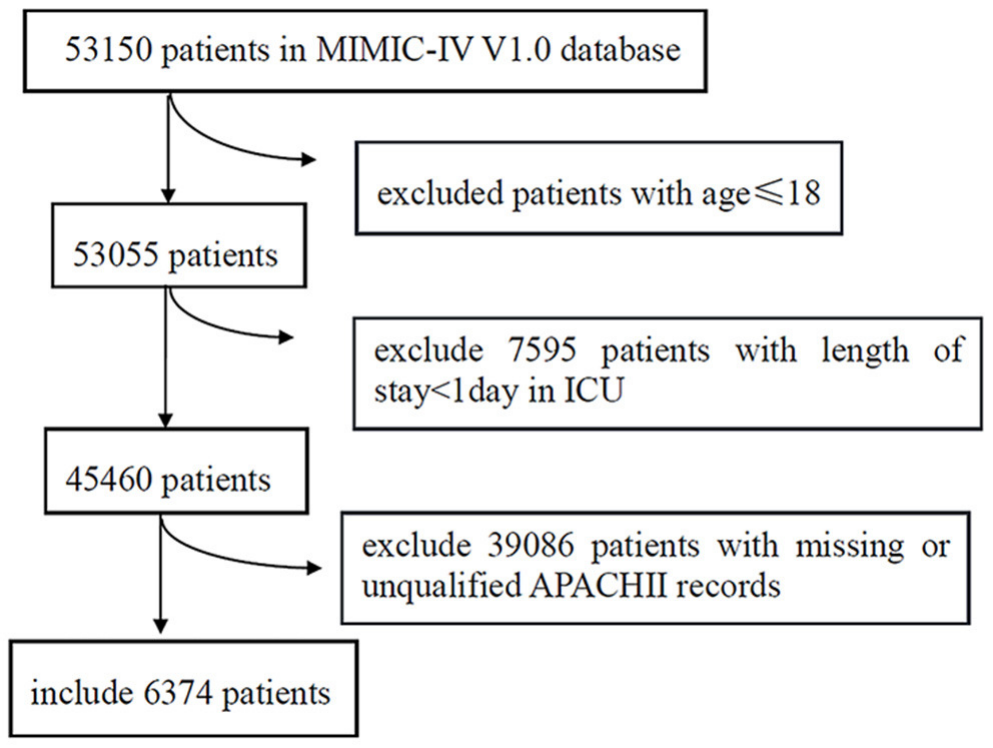
Flow chart of selecting patients according to the inclusion and exclusion criteria.

A total of 6,374 patients in the MIMIC-IV database (53,150 cases) are included in this study according to the inclusion and exclusion criteria (Figure 1). The demographic characteristics, APACHE II scores, source of admission, initial diagnosis, comorbidities, and outcomes of the enrolled patients are summarized in Table 1. The patients are classified into survivors and non-survivors according to their living status. The mean age of the survivors is 63.2 ± 16.7, while the mean age of non-survivors is 68.4 ± 15.9 (*P* < 0.001), suggesting that non-survivors are generally older than the survivors. The percentage of males in the survivors and non-survivors is 58.5% and 54.6 (*P* = 0.013), respectively, suggesting that males may be associated with a lower mortality rate. BMI was 30.5 ± 9.0 kg/m^2^ in the survival group and 28.1 ± 7.5 kg/m^2^ in the non-survival group, The proportion of white, Asian and black people is 69.8, 2.1, and 6.6%, respectively. The Sepsis (including pneumonia) are the most commonly diagnosed disease category among all enrolled patients (*n* = 991, 15.5%). Regarding the spectrum of diseases among the patients, the survival and non-survival groups have a high prevalence of metastatic cancer, distributive shock, and cardica arrest (Table 1). Hypertension is the most common complication among patients (26.5%), followed by heart failure (20.5%) and diabetes (14.2%).

**Table 1:** Demographics, APACHE II score, outcomes and diagnosis of patient.

**Variables**	**All (*n* = 6,374)**	**Survivors (*n* = 5,158)**	**Non-survivors (*n* = 1,216)**	***P*-values**
**Demographics**				
Age, mean (SD), year	64.1 ± 16.8	63.2 ± 16.7	68.4 ± 15.9	*P* < 0.001
Male, No. (%)	3,683 (57.8)	3,019 (58.5)	664 (54.6)	*P* = 0.013
BMI, kg/m^2^	29.6 ± 8.5	30.5 ± 9.0	28.1 ± 7.5	*P* = 0.004
Race and ethnicity				*P < *0.05
White	4,447 (69.8)	3,581 (69.4)	866 (71.2)	
Asian	139 (2.1)	104 (2.0)	35 (2.9)	
Black	421 (6.6)	334 (6.5)	87 (7.2)	
Hispanic/Latino	208 (3.3)	166 (3.2)	42 (3.5)	
Unknown/other	1,159 (18.2)	973 (18.9)	186 (15.2)	
**Source of admission**				*P < *0.05
Ward or step-down unit	956 (15.0)	804 (15.6)	152 (12.5)	
Emergency room	3,187 (50)	2,463 (47.8)	724 (59.5)	
Office or operating room	621 (9.7)	520 (10.1)	101 (8.3)	
Transferred from other hospital	1,575 (24.7)	1,338 (25.9)	237 (19.5)	
Other	35 (0.6)	33 (0.6)	2 (0.2)	
**Comorbidities No. (%)**				
Hypertension	1,689 (26.5)	1,120 (21.7)	569 (46.8)	*P < * 0.049
Diabetes	907 (14.2)	478 (10.0)	429 (35.3)	*P* = 0.062
Liver disease	535 (8.4)	278 (5.4)	257 (21.1)	*P* = 0.047
Chronic kidney disease	637 (10.0)	316 (6.1)	321 (26.4)	*P* = 0.015
COPD	630 (9.9)	389 (7.5)	241 (19.8)	*P =* 0.071
Heart failure	1,306 (20.5)	774 (15.0)	532 (43.8)	*P =* 0.048
Stroke	529 (8.3)	308 (6.0)	221 (18.2)	*P =* 0.059
Anemia	461 (7.2)	274 (5.3)	187 (15.4)	*P =* 0.032
**Initial Diagnosis No. (%)**				
Sepsis (including pneumonia)	991 (15.5)	670 (13.0)	321 (26.4)	*P <0.001*
Non-pulmonary sepsis	507 (8.0)	402 (7.8)	105 (8.6)	*P <0.001*
AKI stage, N (%)				*P* < 0.001
Stage 1	278 (4.4)	266 (5.1)	12 (1.0)	
Stage 2	387 (6.1)	339 (6.6)	48 (3.9)	
Stage 3	157 (2.5)	121 (2.5)	36 (3.0)	
Acute respiratory distress syndrome				*P* < 0.001
Mild	196 (3.1)	162 (3.2)	34 (2.8)	
Moderate	299 (4.7)	224 (4.3)	75 (6.2)	
Sever	150 (2.3)	77 (1.5)	73 (6.0)	
Shock				*P* < 0.001
Hypovolemic shock	221 (3.4)	194 (3.8)	27 (2.2)	
Distributive shock	387 (6.1)	321 (6.2)	66 (5.4)	
Cardiogenic shock	218 (3.4)	183 (3.5)	35 (2.9)	
Obstructive shock	172 (2.7)	145 (2.8)	27 (2.2)	
Metastatic cancer	447 (7.0)	412 (7.9)	35 (2.9)	*P < 0.01*
Acute Respiratory disease	857 (13.4)	810 (15.7)	47 (3.9)	*P < 0.01*
Cardica arrest	396 (6.2)	258 (5.0)	138 (11.3)	*P < 0.01*
Acute poisoning	234 (3.7)	197 (3.8)	37 (3.0)	*P < 0.01*
MODS	260 (4.1)	174 (3.4)	86 (7.1)	*P < 0.01*
Trauma	128 (2.0)	116 (2.2)	12 (1.0)	*P < 0.01*
Others	89 (1.4)	87 (1.7)	2 (0.2)	*P < 0.01*
**Outcomes**				
Hospital mortality, No. (%)	1,216 (19.1)	0	1,216 (100)	*P* < 0.001
ICU mortality, No. (%)	1,001 (15.7)	0	1,001 (82.3)	*P* < 0.001
90-day mortality, No. (%)	1,648 (25.8)	0	1,648 (135.5)	*P* < 0.001
ICU LOS, median (IQR), d	8.62 ± 9.0	8.5 ± 9.1	9.3 ± 8.9	*P* = 0.005
Hospital LOS, median (IQR), d	15.7 ± 13.7	16.4 ± 13.8	12.6 ± 12.8	*P* < 0.001
Mechanical ventilation, No. (%)	829 (13)	461 (8.9)	368 (30.3)	*P* < 0.05
Length of mechanical ventilation (h)	23.13 ± 8.12	20.76 ± 7.13	216.8 ± 18.13	*P* < 0.001
**APACHII score (SD)**				
APACHII on day 1 (*N* = 6,374)	18.4 ± 6.3	18.0 ± 6.3	19.8 ± 6.1	*P* < 0.001
APACHII on day 2 (*N* = 6,374)	15.4 ± 5.8	14.5 ± 5.4	19.6 ± 5.7	*P* < 0.001
APACHII on day 3 (*N* = 4,173)	15.9 ± 5.9	14.5 ± 5.2	20.2 ± 5.8	*P* < 0.001
APACHII on day 5 (*N* = 2,452)	16.8 ± 5.8	14.9 ± 4.9	20.6 ± 5.7	*P* < 0.001
APACHII on day 7 (*N* = 1,835)	17.5 ± 6.3	15.0 ± 5.1	21.6 ± 5.9	*P* < 0.001
APACHII on day 14 (*N* = 858)	17.6 ± 6.6	14.2 ± 4.8	22.4 ± 5.8	*P* < 0.001
APACHII on day 28 (*N* = 364)	15.7 ± 6.9	12.4 ± 4.4	23.2 ± 5.6	*P* < 0.001

*APACHE II, Acute Physiology and Chronic Health Evaluation II; SD, standard deviation; ICU, intensive care unit; LOS, length of stay; IQR, interquartile range*.

**Figure 2:**
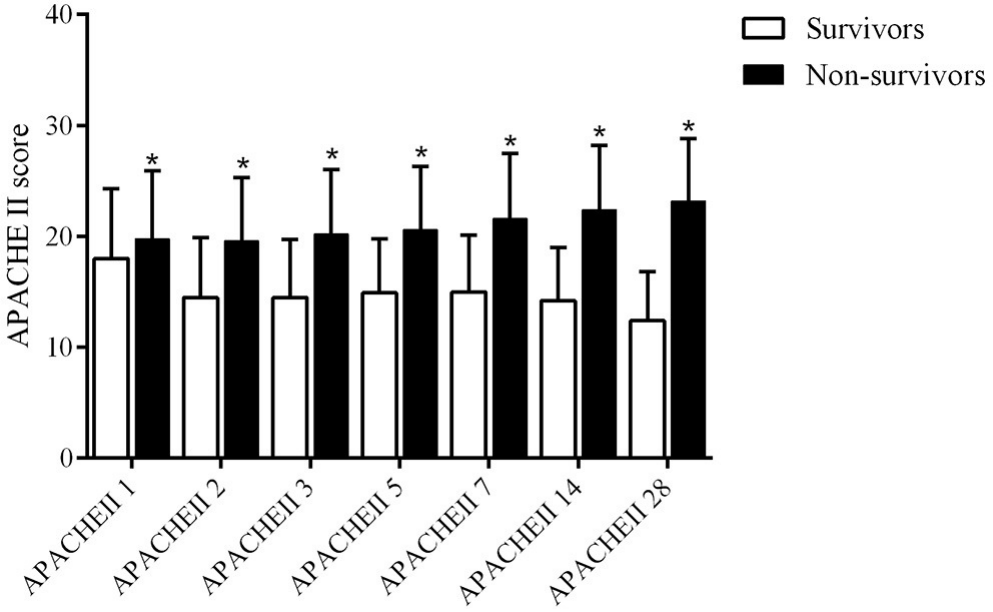
APACHE II score on days 1, 2, 3, 5, 7, 14, and 28 days in survival group and non-survivors group (*P < 0.05).

The APACHE II score of survivors and non-survivors on days 1, 2, 3, 5, 7, 14, and 28 are 18.0 ± 6.3 vs. 19.8 ± 6.1, 14.5 ± 5.4 vs. 19.6 ± 5.7, 14.5 ± 5.2 vs. 20.2 ± 5.8, 14.9 ± 4.9 vs. 20.6 ± 5.7, 15.0 ± 5.1 vs. 21.6 ± 5.9, 14.2 ± 4.8 vs. 22.4 ± 5.8, 12.4 ± 4.4 vs. 23.2 ± 5.6, respectively (all *P* < 0.001, Table 1 and Figure 2). The non-survivor group has higher APACHE II scores than the survivor group at all time points. The survivors have the highest APACHE II score on the first day and the lowest score on day 28, while the APACHE II score of the non-survivors on day 1–day 28 gradually increases, indicating that the non-survivors persistently have more severe diseases and a higher risk of death during hospitalization. The hospital mortality, ICU mortality, and 90-day mortality rates of the non-survivor groups were 100, 82.3, and 25.8%, respectively. The length of stay (LOS) in hospital of the survivors and non-survivors is 16.4 ± 13.8 vs. 12.6 ± 12.8 (*P* < 0.001) days; the ICU LOS of the survivors and non-survivors is 8.5 ± 9.1 vs. 9.3 ± 8.9 (*P* = 0.005) days (Table 1).

There are 829 patients receiving mechanical ventilation, and the length of mechanical ventilation in the non-survival group was higher than that in the survival group (216.8 ± 18.13 vs. 20.76 ± 7.13, *P* < 0.001).

### APACHE II Score in Predicting Hospital Mortality

**Figure 3:**
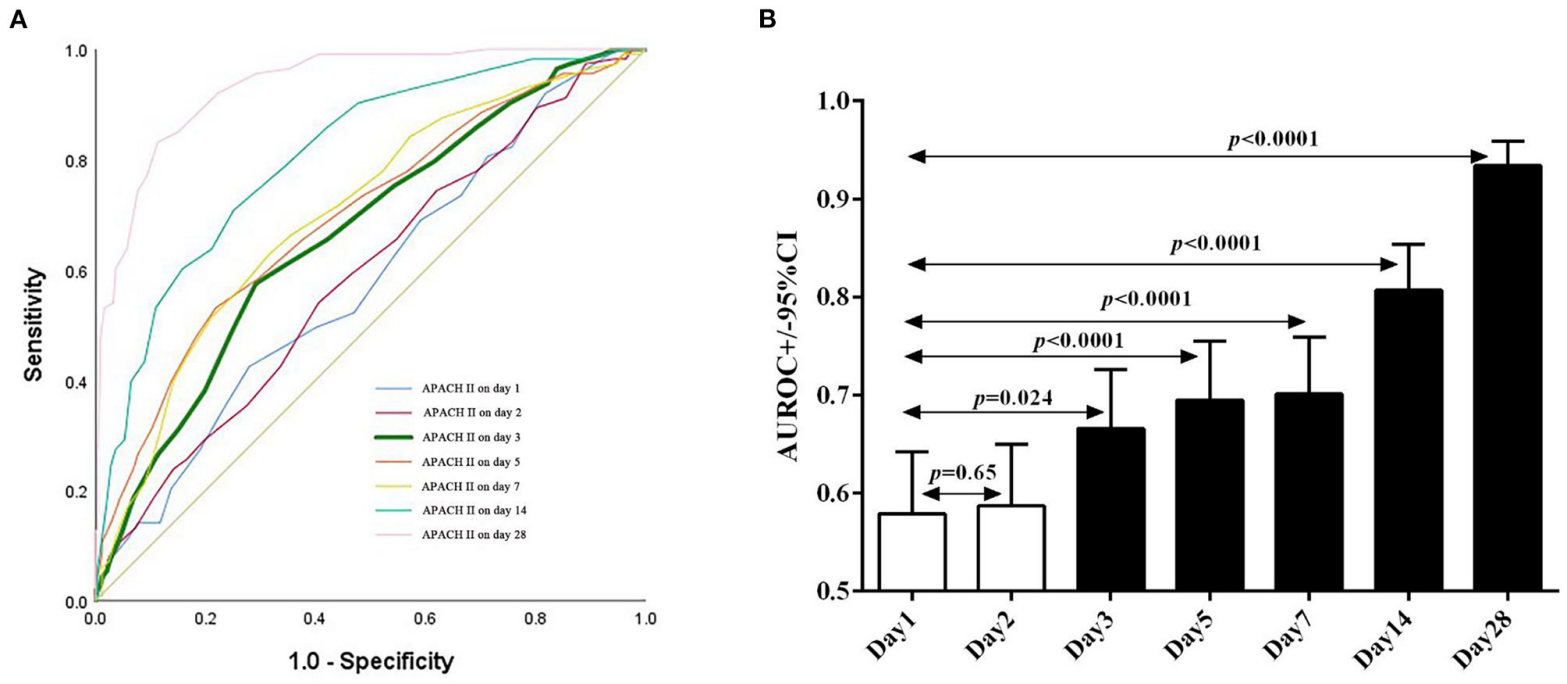
Sequential APACHE II score as predictors of the hospital mortality of patients. **(A)** The receiver operating characteristic (ROC) curves of APACHE II score on days 1, 2, 3, 5, 7, 14, and 28 after admission to ICU. **(B)** The AUROC of APACHE II score on days 2, 3, 5, 7, 14, and 28 is compared to that of day 1.

We then evaluated the performance of the APACHE II score on days 1, 2, 3, 5, 7, 14, and 28 in predicting hospital mortality by measuring the areas under the ROC curves (Figure 3A). The AUROC of each time point is: 0.579 (95% CI: 0.516–0.642), 0.587 (95% CI: 0.524–0.650), 0.666 (95% CI: 0.607–0.726), 0.695 (95% CI: 0.636–0.755), 0.701 (95% CI: 0.643–0.759), 0.807 (95% CI: 0.759–0.854) and 0.934 (95% CI: 0.909–0.959), respectively. Day 3 is the earliest time point whose AUROC is significantly greater than that of the previous days in predicting hospital mortality (*P* = 0.024, Figure 3B). Because early evaluation and intervention are critical in reducing the mortality of ICU patients, we used the APACHE II on day 3 in subsequent analyses, although the AUROC gradually increases as the duration of hospitalization is elongated. We also run serial evaluation of APACHE II score in predicting hospital mortality for the patients who were not transferred from other units or hospitals by ROC curves, The AUROC of day 1, day 2 and day 3 is: 0.496 (95% CI: 0.393–0.598), 0.498 (95% CI: 0.398–0.598), and 0.586 (95% CI: 0.488–0.684), Day 3 is the earliest time point whose AUROC is significantly greater than that of the previous days in predicting hospital mortality (*P* = 0.042) (Supplementary Figure).

### The Performance of APACHE II Score on Day 3 in Predicting Patient Outcomes

**Figure 4:**
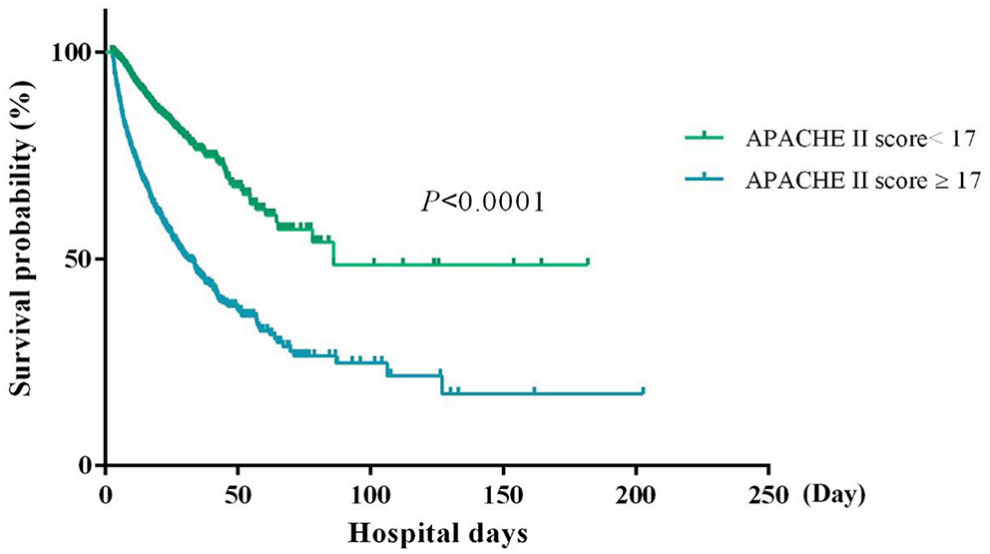
The Kaplan-Meier survival curve of patients with an APACHE II score ≥ or <17.

Since the main usage of the APACHE II scoring system is to identify patients at higher risk of mortality, we used the Youden's index to determine a cut-off score for defining the high mortality risk. We found that score 17 corresponds to the maximum Yorden's index of the day 3 APACHE II ROC. Furthermore, the Kaplan-Meier survival analysis shows that patients with a day-3 APACHE II score >17 have a significantly lower survival rate than those having a score <17 (*P* < 0.0001, Figure 4), further indicating that 17 is an optimal cut-off to distinguish patients with a high or low risk of mortality.

We then evaluated the accuracy of the day 3 APACHE II score in predicting the hospital and 90–day mortality in patients (Table 2). For hospital mortality, In the high-risk patients (APACHE II ≥17, *n* = 4,009), 928 (23.1%) patients are in the non-survival group, the sensitivity is 92.8%, and the positive predictive value (PPV) is 23.1%; 3,081 (76.9%) patients are in the survival group, and the sensitivity is 82.2%. In the low-risk group (APACHE II <17, *n* = 2,365), 288 patients are non-survivors, and the specificity is 90.1%; 2,077 patients are survivors, the specificity is 80.2%, and the negative predictive value (NPV) is 87.8%. For 90-day mortality, in the APACHE II ≥17 group (*n* = 4,009), 1,213 (30.3%) patients are in the non-survival group, the sensitivity is 92.2%, and the PPV is 30.3%; 2,796 (69.7%) patients are in the survival group, and the sensitivity is 81.3%. In the group of APACHE II <17 (*n* = 2,365), 288 (18.4%) patients are non-survivors and the specificity is 100.0%; 1,930 (81.6%) patients are survivors, the specificity is 80.2%, and the NPV is 81.6%.

**Table 2:** Prognostic performance of the third-day APACHE II in hospital mortality.

**APACHE II score**	**Hospital mortality**		**90-day mortality**		
	**Non-survivor (*n*)**	**Survivor (*n*)**	**Non-survivor (*n*)**	**Survivor (*n*)**	**Total**
APACHE II ≥17	928	3,081	1,213	2,796	4,009
	Sensitivity: 92.8%	Sensitivity: 82.2%	Sensitivity: 92.2%	Sensitivity: 81.3%	
	PPV: 23.1%		PPV:30.3%		
APACHE II <17	288	2,077	435	1,930	2,365
	Specificity: 90.1%	Specificity: 80.2%	Specificity: 100%	Specificity: 80.2%	
		NPV: 87.8%		NPV: 81.6%	

*APACHE II, Acute Physiology and Chronic Health Evaluation II; PPV, positive predictive value; NPV, negative predictive value*.

These results suggest that the APACHE II score with a cut-off of 17 has high sensitivity and specificity in predicting the hospital and 90–day mortality of patients.

### Calibration of the APACHE II Score in Predicting 90-Day Mortality

The Hosmer-Lemeshow goodness-of-fit test was then performed to evaluate the calibration of APACHE II score predicting hospital mortality on day 1 and day 3 (Table 3). The results indicate that there is no significant difference between the predicted mortality and the actual mortality (*X*^2^ = 6.198, *P* = 0.625) and the consistency of predicted mortality rate and the actual rate is 79.4%, suggesting that the day-3 APACHE II score-based predictive model has a good calibration ability to predict the hospital mortality. However, APACHE II score on day 1 has a poor calibration in predicting the hospital mortality rate of patients (*X*^2^ = 294.898, *P* < 0.001) (Table 3).

**Table 3:** The Hosmer-Lemeshow goodness of fit test.

**Decline of Risk**	**Survival**		**Non-survival**	
**Hospital mortality**	**Observed**	**Expected**	**Observed**	**Expected**	**Total**
**APECHE II score on day 3**					
1	341	341.392	14	13.608	355
2	327	330.766	28	24.234	355
3	329	321.526	26	33.474	355
4	318	311.866	37	43.134	355
5	302	299.902	53	55.098	355
6	275	284.631	80	70.369	355
7	269	266.116	86	88.884	355
8	239	244.417	116	110.583	355
9	207	211.180	148	143.820	355
10	148	143.203	206	210.797	354
**APECHE II score on day 1**					
1	565	581.870	65	48.130	630
2	562	561.981	68	68.019	630
3	563	548.176	65	81.824	630
4	534	535.885	96	94.115	630
5	542	524.260	88	105.740	630
6	519	512.451	111	117.549	630
7	517	499.186	113	130.814	630
8	488	483.031	142	146.969	630
9	426	458.489	204	171.511	630
10	382	394.670	251	238.330	633

*APACHE II, Acute Physiology and Chronic Health Evaluation II*.

### Adjusted for Other Influence Factor

We verified the confounding factors hospital day, ICU day, and MV day. Model 1 was adjusted for hospital day. Model 2 adjusted for Mode l plus ICU day, Model 3 adjusted for Model 2 plus MV day. After adjusted for all these covariates, APACHE II score on day 3 were significantly correlated with hospital mortality (OR, 1.312, 95% CI, 1.142-1.583). The results confirmed that the model is stable under the influence of the above factors on day 3 (Supplementary Table).

## Discussion

This study focuses on the relationship between APACHE II scores and the outcomes of ICU patients. We found that APACHE II score on day 3 is the optimal predictor of the outcomes in ICU patients.

A large number of studies have confirmed that the APACHE II score is a useful prognostic biomarker of the mortality of patients with a critical illness. A retrospective study of 200 Iranian ICU patients reported that an APACHE II score of 15 provides the best accuracy to predict the mortality of critically ill patients (6). This study indicated that APACHE II score of 17 is an optimal cut-off to distinguish patients with a high or low risk of mortality. The difference in results may be attributed to different sources of patients. Liu et al. have shown that the initial APACHE II scores on the day of ICU admission are correlated with the outcomes of patients (16). A study that included 109 cirrhotic MICU patients reported that APACHE II could be used as a predictor of mortality (17). Notably, most currently available studies used APACHE II score within 24 h after admission, which is helpful in classifying patients and early identifying risk factors. However, some factors affecting the prognosis of ICU patients within 24 h may not be included in the APACHE II score system, resulting in inaccurate predictions of patients' outcomes. This study found that the first-day APACHE II score has a poor calibration on hospital mortality of the included cohort of patients. Kim et al. (8) also found that the APACHE II of the first 24 h after admission to the ICU exhibits poor calibration for hospital mortality in a study including 826 Korean patients. In another large-scale study including 141,106 ICU patients in the U.K., the APACHE II score showed good discrimination but imperfect calibration for hospital mortality (18). Yoon et al. (19) observed that The APACHE II score on day 3 had the highest prognostic value for predicting poor neurologic outcomes with an area under the cure of 0.793, and with a cut-off value of 20, the APACHE II score predicted poor neurologic outcomes with a sensitivity of 43.75%, a specificity of 94.12%, a positive predictive value of 94.59%, and a negative predictive value of 41.56%. However, The subjects of their study were survivors of hospital cardiac arrest, and the APACHE II score was used to evaluate the prognosis of the nervous system.While in our study, the APACHE II scores at different time points were used to evaluate hospital mortality of ICU patients. Yoon's study and our study are performed among different subjects with different aims and end-points. Other study performed by Donnino et al. (20) also observed the same in the cardiac arrest population and concluded that The discrimination for APACHE Score II score was best at the 72-h mark after cardiac arrest.Similar to Yoon's study, Donnino's study also applied the APACHE II score in patients with post-cardiac arrest to evaluate the role of the scoring system in discriminating cardiac arrest. This study and our study are performed on different subjects with different aims.Therefore, a better prognostic biomarker is necessary to accurately predict the mortality of ICU patients. In recent years, several new models have been developed to predict the mortality of ICU patients (21), which showed certain values in clinical practice. However, the APACHE II score is still the most widely accepted model in predicting the mortality of critically ill patients. Therefore, further development of the APACHE II model, such as finding an optimal time point other than 24 h for calculating the score by comprehensively analyzing multicenter and large-scale studies, is still an effective way to improve the accuracy of the APACHE II system.

The dynamic SOFA score has been shown to obtain higher accuracy in predicting the outcome of patients with sepsis than the fixed-day SOFA score (7, 22, 23). Similarly, some researchers suggest that the APACHE II score should be calculated daily during the initial seven days of ICU stay to reduce the error to the greatest extent (24). However, due to the complexity of calculating the APACHE II score, the daily assessment will result in a huge workload for the caregivers, so it is more practical to identify the optimal time point to calculate the APACHE II score that best predicts the outcome of ICU patients. Based on this idea, serial APACHE II scores were calculated on days 1, 2, 3, 5, 7, 14, and 28 after admission to ICU in this study. We found that the third-day APACHE II score is the optimal predictor of the hospital and 90-day mortality in ICU patients included in this study.

The cut-off of the APACHE II scores that provides the best accuracy in predicting the mortality of patients still has controversy. Bahtouee et al. have reported that an APACHE II score of 15 gave the best accuracy to predict ICU mortality (6). However, another two studies reported that the best cut-off score for APACHE II in predicting hospital mortality was 13.5. In this study, we discovered that 17 is the best cut-off for the third-day APACHE II score. The variations in the cut-offs among studies may stem from the differences in time points for calculating the APACHE II score and sample size of the studies. First, some important factors influencing the survival of patients may be changed after the first 24 h of admission to ICU due to resuscitation and other therapies (25, 26), resulting in the changes in the performance of APACHE II in predicting the mortality of patients. The previous study was based on the first-day APACHE II score, while our study applied the third-day APACHE II score. Therefore, different cut-offs have to be applied to make sure the best performance of APACHE II at each time point. Second, the sample size of the previous studies is usually small, but this study is based on a much larger cohort of patients.

There are several limitations of this study. This is a retrospective study, which is prone to have selection bias. However, all the necessary data have been collected when we retrieve the data from the MIMIC-IV dataset to avoid possible bias. Additionally, most of the patients included in the MIMIC-IV study are treated in North American ICUs. Therefore, the conclusion may not be directly applied to ICUs in other locations in the world. More studies need to be done to evaluate the conclusion in other countries. Finally, our study only included data available online, and more external validation is still required.

APACHE II is the most widely used and authoritative critical illness evaluation system in intensive care unit. It can objectively formulate and revise the medical care plan by evaluating the condition and predicting the mortality of patients in ICU, and provides an objective and scientific basis for improving medical quality, making rational use of medical resources and determining the best time of discharge or selecting the time of treatment. Therefore, if the mortality can be accurately predicted, corresponding medical measures can be taken to improve the mortality to a certain extent. Compared with the traditional APACHE II score on the first day, this study find that the APACHE II score on the third day is more helpful to accurately distinguish the condition of critically ill patients, Therefore, corresponding medical monitoring and personalized treatment strategies can be adopted according to the APACHE II score on the third day to improve the mortality of patients. However, clinical judgment will still require integration of wide range of medical facts. These results are only regarded as auxiliary indicators of medical decision-making. Further multicenter large sample size prospective studies are needed to verify the accuracy of the third day APACHE II score in predicting patient mortality in more critically ill patient populations.

## Conclusion

APACHE II score on day 3 with a cut-off of 17 is the optimal biomarker to predict the outcomes of ICU patients. This finding will provide a basis for medical staff to adopt appropriate medical strategies.

## Data Availability Statement

The original contributions presented in the study are included in the article/Supplementary Material, further inquiries can be directed to the corresponding author.

## Author Contributions

SW obtained funding for the study. YT and YY proposed the initial study concept and design and performed data collection and analysis. JZ, XD, and HC contributed to the final study design. KC and XM participated in statistical analysis and provided valuable comments on the manuscript. YT, YY, and SW drafted the manuscript. All authors contributed to the critical revision for the intellectual content and read and approved the final manuscript.

## Funding

This project was supported by funds from the Seventh Batch of Science and Technology Planning Projects in Xi'an [20YXYJ0001 (07)] and Basic and transformation research innovation team of respiratory and critical diseases in Xi'an Medical College.

## Conflict of Interest

The authors declare that the research was conducted in the absence of any commercial or financial relationships that could be construed as a potential conflict of interest.

## Publisher's Note

All claims expressed in this article are solely those of the authors and do not necessarily represent those of their affiliated organizations, or those of the publisher, the editors and the reviewers. Any product that may be evaluated in this article, or claim that may be made by its manufacturer, is not guaranteed or endorsed by the publisher.

## Supplementary Material

Supplementary Figure Sequential APACHE II score as predictors of the hospital mortality of patients who were not transfered from other units or hospitals.

Supplementary Table Odds Ratios and 95% CIs for Hospital Mortality Associated With the APACHE II score in Overall Patients after adjusted for hospital day, ICU day, and MV day.

## Abbreviations

APACHE II: The Acute Physiology and Chronic Health Evaluation II
AUROC: area under the receiver operating characteristic
SOFA: Sequential Organ Failure Assessment
SAPS: Simplified Acute Physiology Score
MIMIC: Medical Information Mart for Intensive Care
LOS: length of stay
ICD-9: International Classification of Diseases-9th.
