# Supplementary figures and images for: Dynamic APACHE II Score to Predict the Outcome of Intensive Care Unit Patients
Source: Front Med (Lausanne). 2022 Jan 26;8:744907. doi: 10.3389/fmed.2021.744907 (PMC8826444; doi:10.3389/fmed.2021.744907)

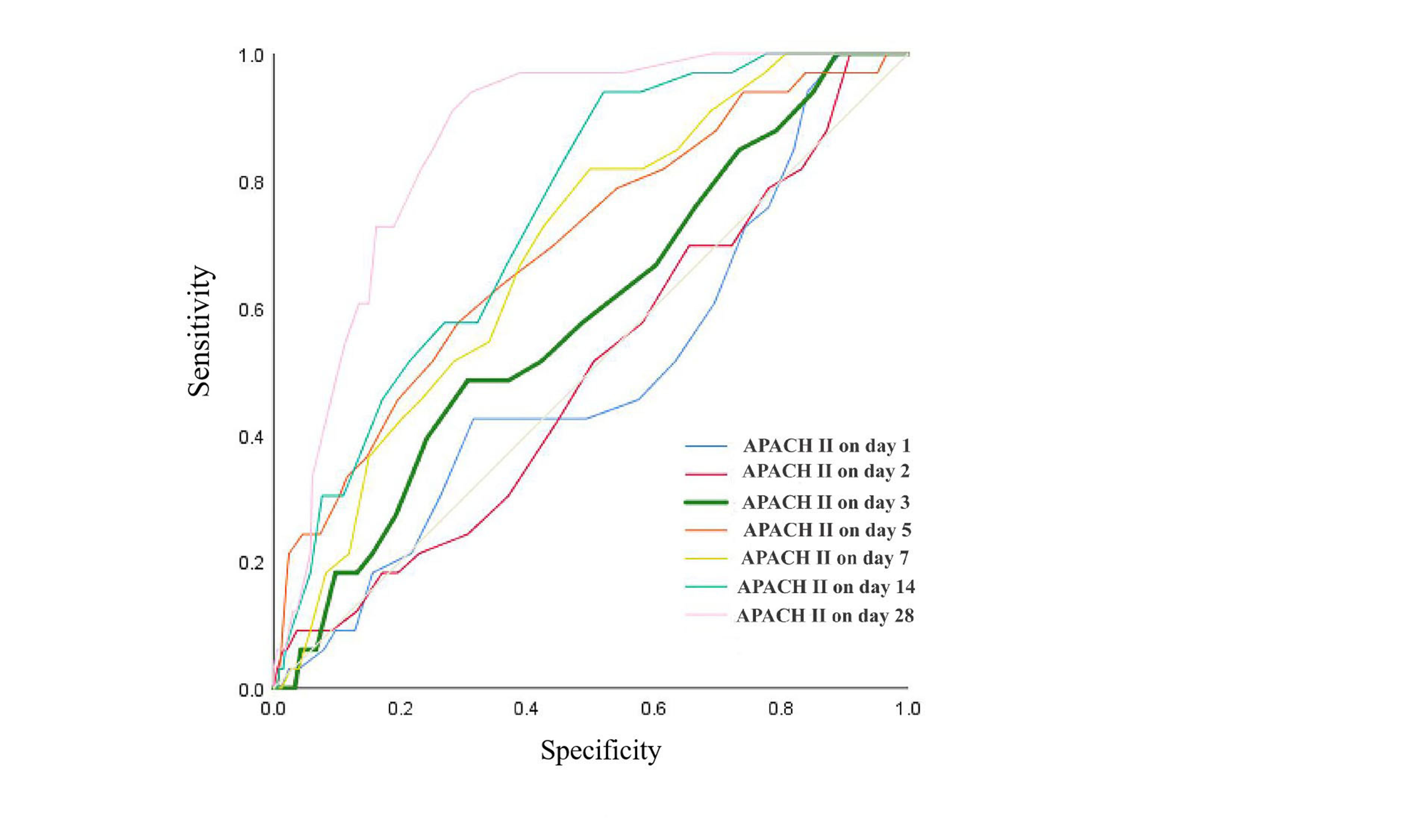

Supplement: Supplementary Figure — Sequential APACHE II score as predictors of the hospital mortality of patients who were not transfered from other units or hospitals. [file Image_1.JPEG]
