# Supplementary material for: Dynamic APACHE II Score to Predict the Outcome of Intensive Care Unit Patients
Source: Front Med (Lausanne). 2022 Jan 26;8:744907. doi: 10.3389/fmed.2021.744907 (PMC8826444; doi:10.3389/fmed.2021.744907)
Supplement: Supplementary Table — Odds Ratios and 95% CIs for Hospital Mortality Associated With the APACHE II score in Overall Patients after adjusted for hospital day, ICU day, and MV day. [file Table_1.DOC]

Supplemental table: Odds Ratios and 95% CIs for Hospital Mortality Associated With the APACHE II score in Overall Patients after adjusted for hospital day, ICU day, and MV day

|  | OR | 95%CI) | *P* |
| --- | --- | --- | --- |
| Model 1 | 1.436 | 1.245-1.672 | <0.001 |
| Model 2 | 1.367 | 1.162-1.528 | <0.001 |
| Model 3 | 1.312 | 1.142-1.583 | <0.001 |
